# Structural morphometry distinguishes vagus nerve stimulation outcomes in individuals with epilepsy

**DOI:** 10.1002/epi4.70339

**Published:** 2026-08-31

**Authors:** Harry J. Clifford, Sonja Fenske, Yujiang Wang, Tiago da Silva Costa, Rhys H. Thomas, Sabahat Iqbal, Cameron A. Elliott, Davide Giampiccolo, John S. Duncan, Peter N. Taylor

**Affiliations:** ^1^ CNNP Lab (www.cnnp‐lab.com), Computational Medicine Group School of Computing, Newcastle University Newcastle upon Tyne UK; ^2^ Faculty of Medical Sciences Newcastle University Newcastle upon Tyne UK; ^3^ UCL Queen Square Institute of Neurology, Queen Square London UK; ^4^ Northern Centre for Mood Disorders Newcastle University; Cumbria, Northumberland, Tyne and Wear NHS Foundation Trust Newcastle upon Tyne UK; ^5^ National Institute for Health and Care Research (NIHR) Newcastle Biomedical Research Centre Newcastle upon Tyne UK; ^6^ Neurosciences, Royal Victoria Infirmary Newcastle‐upon‐Tyne UK; ^7^ Department of Brain Sciences Imperial College London London UK; ^8^ Division of Neurosurgery, Department of Surgery University of Alberta Edmonton Alberta Canada

**Keywords:** drug resistant epilepsy, structural MRI morphometry, treatment response prediction, vagus afferent network, vagus nerve stimulation

## Abstract

**Objective:**

Vagus nerve stimulation (VNS) is a commonly used treatment for drug‐resistant epilepsy. However, response to VNS is variable, while understanding of this response variability is limited. The vagus afferent network (VagAN), the neural circuits thought to be involved in response to vagal afferents, has been implicated in response prediction in modalities such as diffusion‐weighted imaging, functional imaging, and neurophysiology. Structural abnormality in the VagAN from T1w MRI has not yet been studied.

**Methods:**

We assessed if VagAN abnormalities, derived from pre‐implantation T1w MRI, were associated with VNS response in 92 individuals with epilepsy two years post‐implantation (42 responders, 50 non‐responders). We used the first principal component (PC1) from a robust principal component analysis for a variety of region sets trained using 100 healthy controls. We investigated two primary hypotheses. (i) If VagAN regions' PC1 differentiates response groups, (ii) if other region sets did not differentiate response groups.

**Results:**

Non‐responders had significantly higher VagAN PC1 abnormality scores than both responders (r=0.30, p=0.008) and controls (r=0.37, p<0.005). By contrast, non‐VagAN PC1 showed no discriminatory value (r=−0.03, p>0.05). VagAN outperformed all other region sets.

**Significance:**

Structural abnormality of VagAN was associated with non‐response to VNS. This suggests that volume abnormalities in key VNS regions correlate negatively with reduction of seizures. Abnormalities in other regions, such as the non‐VagAN region set, were not associated with VNS response. This metric, when combined with other modalities, may be a useful measure to improve the selection of individuals with drug‐resistant epilepsy for VNS therapy.

**Plain Language Summary:**

Vagus nerve stimulation (VNS) is a common treatment for drug‐resistant epilepsy; however, it is not always effective. We tested whether volumes of brain regions related to VNS efficacy and found that abnormality within a set of regions known to be affected by VNS related to non‐response. No other set of regions showed this effect implying that this set of regions is uniquely placed to determine if someone may respond to VNS. This work supports the idea that VNS efficacy is limited by an individual's brain abnormality and should be considered in further studies.

**Key points**

1. Response to vagus nerve stimulation (VNS) is variable; however, reasons for this are poorly understood.
2. Non‐responders to VNS show significantly more structural abnormality in the vagus afferent network (VagAN) than responders and controls, based on pre‐implant T1w MRI.
3. This abnormality is specific to the VagAN with no other brain region set, including non‐VagAN, structural, and intrinsic connectivity networks distinguishing response.
4. VagAN structural morphometry from routine T1w MRI could help identify reasons for non‐response prior to VNS implantation with non‐responders demonstrating more abnormal metrics across multiple modalities.

## 1 INTRODUCTION

Vagus nerve stimulation (VNS) is a palliative treatment for drug‐resistant epilepsy (DRE) if neurosurgical resection is not an option. Responders to VNS are traditionally defined as those who achieve a ≥50% reduction in seizure frequency, which occurs in around half of individuals implanted. Although response rates have been associated with early implantation,^1^, ^2^ this has only been demonstrated in pediatric cohorts, and demographic factors alone do not distinguish responders from non‐responders.^3^, ^4^, ^5^, ^6^

Biomarkers of VNS response have been suggested across many clinically acquired modalities,^5^, ^7^ but little attention has been paid to T1‐weighted magnetic resonance imaging (T1w MRI). The routine clinical acquisition of T1w MRI presents a valuable opportunity for further research on this modality. A commonly discussed biomarker is the presence of lesions on T1w MRI,^5^, ^8^, ^9^, ^10^, ^11^, ^12^ but studies examining structural features beyond radiologically defined lesions are limited. Two studies have examined structural features beyond visible lesions: our previous examination of hippocampal abnormality^8^ and an examination of deformation‐based abnormalities.^6^

VNS affects multiple interconnected brain regions. The vagus afferent network (VagAN) has been suggested to comprise areas affected by VNS.^5^, ^13^ VagAN regions are either directly innervated by vagal afferent fibers or receive input via the nucleus tractus solitarius. The VagAN includes amygdala and thalami, abnormalities of which in VNS non‐responders have been supported by findings across multiple modalities.^14^, ^15^, ^16^, ^17^ The VagAN may be key to the therapeutic effect of VNS. To date, no study has examined whether VagAN structural abnormalities on T1w MRI are associated with VNS response.

In this study, we investigated the following questions:

1. Are structural abnormalities on pre‐implant T1w MRI present in the VagAN and do they differ between VNS response groups?
2. Are response‐related structural abnormalities found in other brain regions?

## 2 METHODS

### 2.1 Ethical approval

This study of anonymized data that had been acquired previously was approved by the Health Research Authority, without the necessity to obtain individual subject consent (UCLH epilepsy surgery database: 22/SC/0016), and by the Database Local Data Monitoring Committee. Individuals who declined for their data to be used in anonymized research were not included in the analysis.

### 2.2 Cohort

We retrospectively identified individuals with epilepsy and an implanted VNS from the National Hospital for Neurology and Neurosurgery using clinical electronic records from before July 2022. We excluded individuals without T1w MRI acquired at the Chalfont Centre for Epilepsy prior to implantation or without an ascertainable 2‐year outcome. For the control cohort, healthy participants were also scanned at the Chalfont Centre for Epilepsy. Detailed exclusion criteria are shown in Figure S1. Individual response to VNS was defined as > 50% reduction in seizure frequency at the clinical check‐up closest to 2 years after VNS implantation, extracted from clinical letters. These data have been studied previously.^8^

### 2.3 Demographics

We previously reported the full demographics of the VNS cohort,^8^ with controls described in a prior data release.^18^ Key demographic data are presented in Table 1. In brief, non‐responders were significantly more likely to have a clinically identified lesion on MRI (p=0.037), including hippocampal sclerosis (HS) (p=0.036). We perform our primary analysis including both those with and without lesion presence in the main text. In Supporting Information, we replicate analyses for only individuals with lesions on MRI and analyses for individuals with MRI lesions other than HS.

**TABLE 1:** Demographic information for individuals with DRE and healthy controls.

Demographic	Non‐responders	Responders	Controls	Test statistic
Sex, female:male	33:17	20:22	62:38	*χ* ^2^ = 4.65, *p* = 0.199
Age at scan, median (IQR)	39 (17.8)	32.5 (17.8)	39 (21)	*F* = 2.45, *p* = 0.092
Scan type, FSPGR:MPRAGE	30:20	26:16	30:70	*χ* ^2^ = 18.5, *p* < 0.001
Duration, median (IQR)	28 (18.5)	23 (15.5)	NA	*F* = 1.28, *p* = 0.203
Scan gap, median (IQR)	2.4 (2.5)	3.3 (5)	NA	*F* = −1.86, *p* = 0.066
MRI lesion presence, yes:no	31:18	16:25	NA	*χ* ^2^ = 4.33, *p* = 0.037
Hippocampal sclerosis, yes:no	13:37	3:39	NA	*χ* ^2^ = 4.41, *p* = 0.036
Localization, TLE:ETLE	25:14	13:11	NA	*χ* ^2^ = 0.27, *p* = 0.605

Abbreviations: ETLE, extratemporal lobe epilepsy; FSPGR, Fast spoiled gradient echo; IRQ, interquartile range; MPRAGE, magnetization‐prepared rapid acquisition gradient echo; TLE, temporal lobe epilepsy.

### 2.4 T1w MRI acquisition and region sets

#### 2.4.1 MRI acquisition

T1w MRI scans were acquired at the Chalfont Centre for Epilepsy using one of two 3T GE scanners. Individuals underwent either a fast spoiled gradient echo (FSPGR) scan with a resolution of 0.9375×0.9375×1.1 mm, or a magnetization‐prepared rapid acquisition gradient echo (MPRAGE) scan with a resolution of 1×1×1 mm. Detailed acquisition parameters for control participants have been previously described.^18^

#### 2.4.2 Vagus afferent network (VagAN)

VagAN regions include the amygdala, anterior cingulate cortex (ACC), brainstem, hypothalamus, insula, prefrontal cortex (PFC), and thalamus. We use the VagAN as the primary region set for which we hypothesized non‐responders to demonstrate higher relative abnormality to responders.

#### 2.4.3 Additional region sets

To determine whether the VagAN shows greater discriminatory value than other brain regions, we additionally analyzed other region sets, some of which partially overlap with VagAN (Table S1). Specifically, we investigated whether any of the following region sets outperformed the VagAN (the number of regions in each set is shown in parentheses): cingulate cortex and insula (12), frontal lobe (20), occipital lobe (8), parietal lobe (12), temporal lobe (14), default mode network (10), dorsal attention network (6), language (10), salience (6), sensorimotor (6), somatosensory (6), visual (8), and all regions not in the VagAN (non‐VagAN, 40). Regions included in each region set are available in Table S1, with the overlap with each other and the VagAN shown in Figure S6. These region sets were chosen to represent both structural regions (divided into lobes) and intrinsic connectivity networks.^19^, ^20^ Additionally, we included the non‐VagAN set to evaluate regions which are hypothesized to not be involved in VNS response. The somatosensory network was additionally included based on its previous investigation in VNS response.^16^

#### 2.4.4 Preprocessing

We computed regional volumetry using FreeSurfer (version 7.4.1; https://surfer.nmr.mgh.harvard.edu/) with the Desikan‐Killiany atlas for cortical parcellation. FreeSurfer segmentations were manually inspected for quality, edited with control points, and rerun where necessary. For subcortical parcellations, we used additional FreeSurfer packages for the amygdala,^21^ brainstem,^22^ hypothalamus,^23^ and thalamus.^24^ For the amygdala, hypothalamus, and thalamus, we combined subregional volumes into larger parcels to improve volumetric consistency while maintaining subregional resolution. The following combinations were used, with the combined parcellation name listed first, then after the colon, the subregions from the above atlases that parcellation consists of:

For the amygdala:

1. Lateral nucleus: lateral nucleus.
2. Posterior central: accessory basal nucleus, central nucleus.
3. Summed nuclei: anterior amygdaloid area (AAA), basal nucleus, cortical nucleus, cortico‐amygdaloid transition, medial nucleus, paralaminar nucleus.

For the hypothalamus:

1. Anterior: anterior inferior, anterior superior
2. Posterior: posterior
3. Tubular: tubular inferior, tubular superior

For the thalamus:

1. Anterior lateral: anteroventral, laterodorsal, lateral posterior.
2. Intralaminar: central medial, central lateral, centromedian, paracentral, parafascicular.
3. Medial: paratenial, medial ventral, mediodorsal medial magnocellular, mediodorsal lateral parvocellular.
4. Posterior: lateral geniculate, medial geniculate, limitans (suprageniculate), pulvinar anterior, pulvinar medial, pulvinar lateral, pulvinar inferior.

### 2.5 Abnormality calculation

#### 2.5.1 Principal component analysis (PCA)

Principal component analysis (PCA) is a dimensionality reduction technique commonly used to summarize multivariate data. We specifically used robust PCA^25^, ^26^, ^27^ to reduce the effect of any possible outliers within controls and refer to this as simply “PCA” throughout the manuscript. Volumes were corrected using ComBat to remove any scan‐specific effects (FSPGR or MPRAGE) while retaining differences due to sex, age, and presence of epilepsy.^28^ Volumes were then corrected using a robust linear regression model trained on controls to remove sex, age, and intracranial volume effects before being transformed into z‐scores using control values. PCA was performed on the control group only, with the resulting transformation then applied to all individuals. Specifically, we used the first principal component (PC1), the component explaining the greatest variance in the control population, to reduce each region set to a single value per individual. Limiting our analysis to PC1 discards a large amount of the structural variance but is necessary to reduce the possibility of false positives from multiple tests. Additional components may also contain useful information but are untested here due to the lack of statistical power. We then calculated regional abnormality scores for all individuals (responders, non‐responders, and controls) by computing the absolute z‐score of each individual's PC1 value relative to the control distribution for each region set. Higher absolute z‐scores indicate greater deviation from the typical control pattern, as PCA values do not have clear direction to represent either atrophy or hypertrophy. Throughout this manuscript, we refer to these values as “region set PC1” (e.g., VagAN PC1, non‐VagAN PC1).

### 2.6 Statistical analysis

All data analysis was performed using R version 4.3.2 (https://www.r‐project.org). To test the significance of demographic data, we used $\chi^{2}$ for categorical data and analysis of variance (ANOVA) for numeric data. We used non‐parametric measures as we cannot be certain of a normal distribution amongst our calculated abnormalities. Group comparisons were performed using the Wilcoxon rank‐sum test (Mann–Whitney *U*), and effect sizes were calculated using rank‐biserial correlation (biserial *r*) with corresponding qualitative labels.^29^ We also use Pearson's R and associated *p*‐value to compare correlations between continuous variables. We did not apply multiple‐comparison correction because our hypothesis specifies that VagAN abnormalities should outperform the other region sets. Correcting these comparisons using sets expected a priori to show no effect would therefore be overly conservative. Our primary statistical test (comparing VagAN PC1 abnormality between responders and non‐responders) constitutes a single pre‐specified hypothesis, and as such does not form part of a family of tests requiring correction. The remaining region sets were included as negative controls, selected a priori on the basis that they were not expected to show response‐related differences. Nonetheless, to support transparency, we report FDR‐corrected *p*‐values for all secondary region sets excluding the VagAN and non‐VagAN, acknowledging that any significant findings amongst these sets should be interpreted as exploratory. All tests were also one‐tailed due to our prior hypothesis of greater abnormality in non‐responders, although we note if/when effects differ to this expectation.^5^, ^8^ In supplementary analyses, we also report our findings alongside our previous hippocampal analyses^8^ on the same data.

## 3 RESULTS

The results are organized into two sections.

1. Are structural abnormalities on pre‐implant T1w MRI present in the VagAN, and do they differ between VNS response groups?
2. Are response related structural abnormalities found in other brain regions?

Results of analyses excluding individuals without any clinically identified lesion, as well as analyses specifically excluding individuals with HS are available in the Supporting Information.

### 3.1 VagAN structural abnormalities differ between response groups

To generate a single VagAN abnormality score per individual, we performed PCA on VagAN regional volumes from the control group and extracted the first principal component (PC1). VagAN PC1 explained 36.3% of the variance in controls, with the first six components (PC1–PC6) explaining a cumulative variance of 85% (Figure 1A). VagAN PC1 loadings demonstrated widespread regional involvement across the VagAN (Figure 1B, see Table S2 for detailed loadings and Figure S7 for a visualization).

**FIGURE 1:**
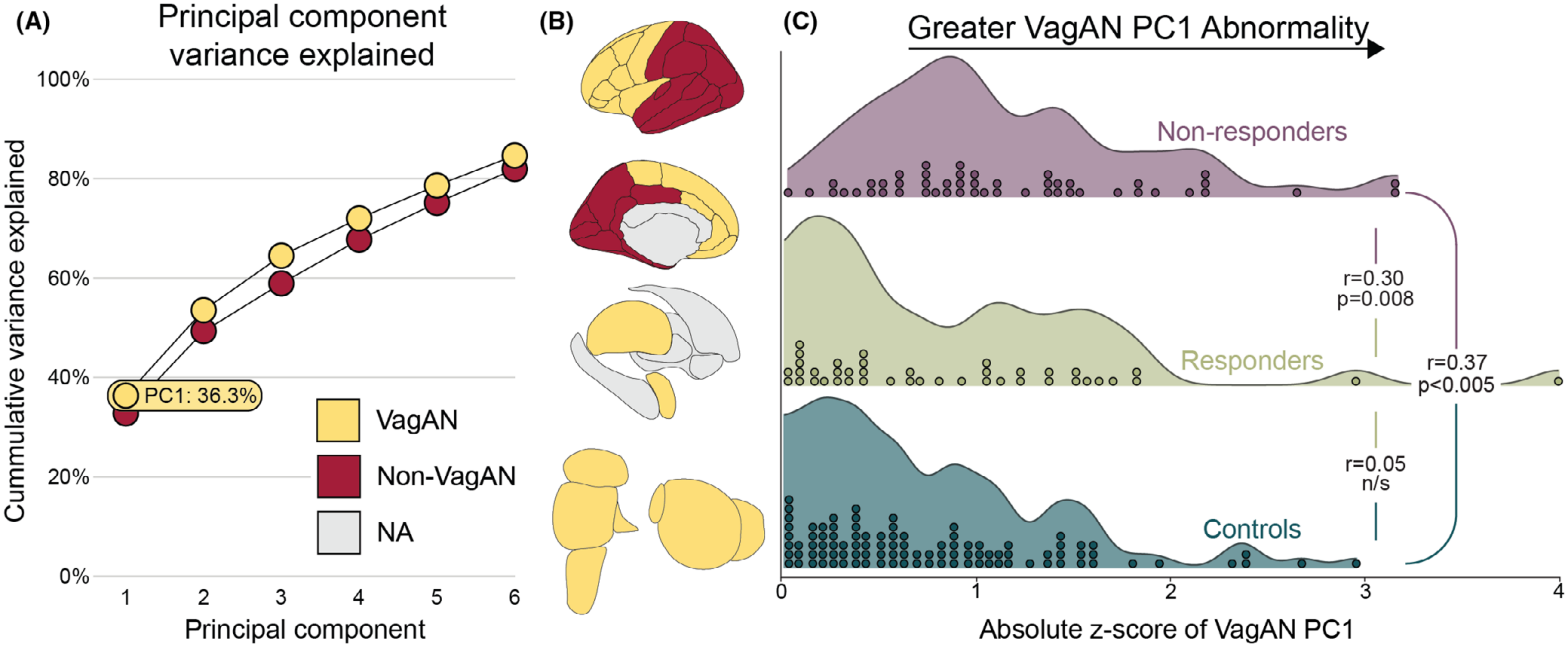
Non‐responders had greater VagAN structural abnormality than responders and controls. (A) Shows the cumulative variance explained by principal components of the VagAN. VagAN PC1 explained 36.3% of the variance in controls, with the subsequent five components (PC2–PC6) explaining an additional 17.2%, 11.0%, 7.5%, 6.6%, and 6.0%, respectively (cumulative variance: 84.6%). (B) Shows the regions contained within the VagAN and outside the VagAN. Exact loadings as well as a visualization on the brain are available in Table S2 and Figure S7 respectively. (C) Non‐responders showed significantly greater VagAN PC1 abnormality (absolute z‐score) than both responders (*r* = 0.30, *p* = 0.008) and controls (*r* = 0.37, *p* < 0.001). Responders did not differ significantly from controls (*r* = 0.05, *p* = 0.33).

We next examined if VagAN PC1 abnormality differed between response groups (Figure 1C). Non‐responders showed significantly greater VagAN PC1 abnormality than both responders (r=0.30, p=0.008) and controls (r=0.37, p<0.001). Responders did not differ significantly from controls (r=0.05, p=0.33). Mass univariate testing of individual VagAN regions did not significantly differentiate response groups (Table S3).

### 3.2 VagAN showed greater discriminatory value than other region sets

The PC1 of each region set explained between 32.8% and 72.1% of the variance in controls. The variance explained by PC1 was negatively correlated with the number of regions in each set, as larger region sets captured more dimensions of structural variation (R=−0.51 for all regions, R=−0.57 when excluding VagAN and non‐VagAN, Figure S2). There was no correlation between effect size seen between responders and non‐responders and number of variance explained by PC1 (R=−0.032, p=0.91). Exact PCA variance and VagAN overlap for all region sets are provided in Table S4.

VagAN PC1 showed the greatest discriminatory value between response groups compared with all other region sets (Figure 2). No other region set showed significant differentiation between responders and non‐responders after multiple‐comparison correction. Combined non‐VagAN regions showed no significant difference between response groups (r=−0.03, p=0.78). When excluding individuals with clinically identified HS only VagAN PC1 remained significant. Effect sizes were slightly reduced for both VagAN and language networks but increased for the DAN (Figure S3).

**FIGURE 2:**
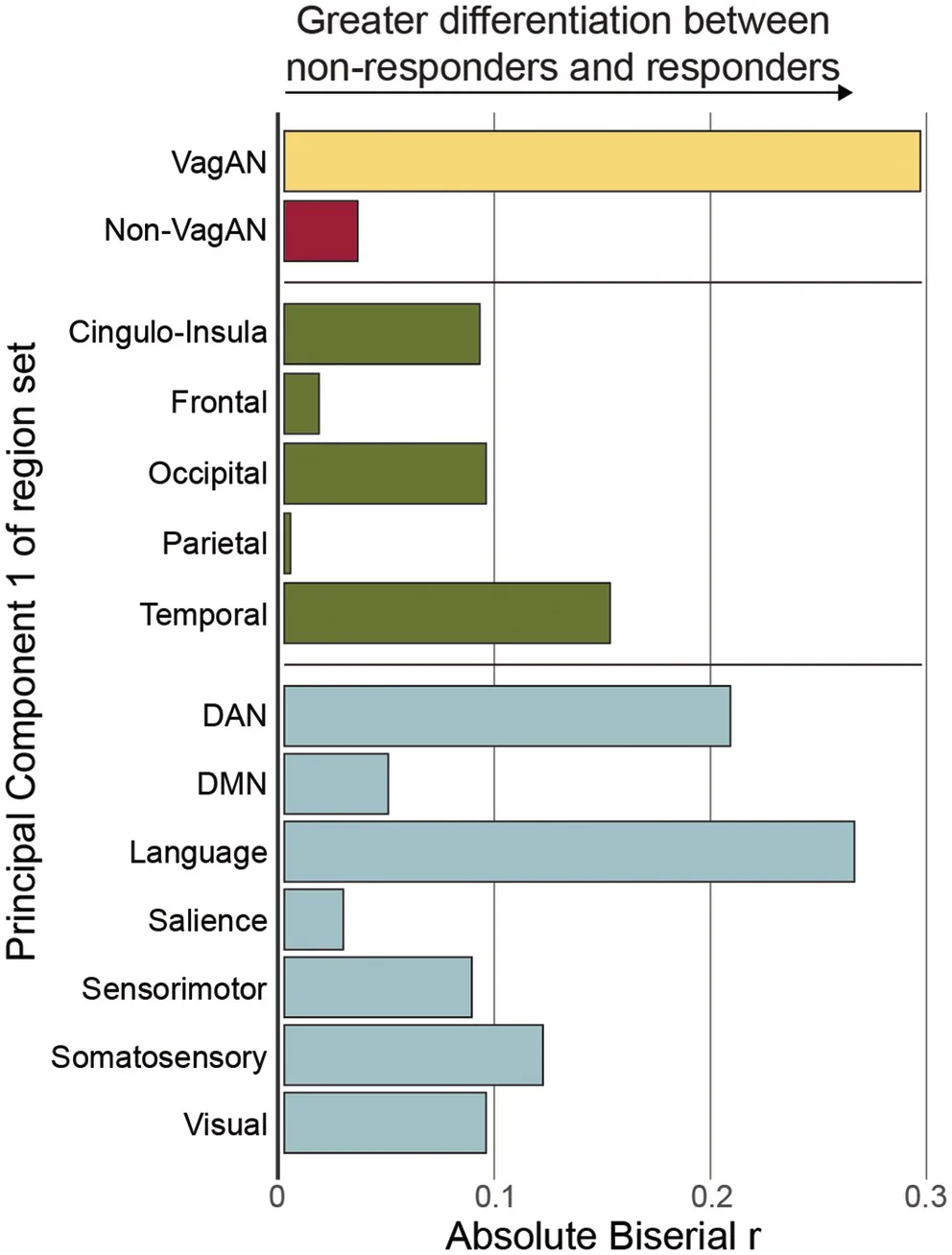
VagAN shows greater discriminatory value than all other region sets. VagAN PC1 significantly differentiated non‐responders from responders (*r* = 0.30, *p* = 0.008). The combined non‐VagAN region set showed no discriminatory value (*r* = −0.03, *p* = 0.78), and structural region sets based on lobular anatomy (seen in green) did not significantly differentiate response groups (all *p* > 0.20). Across connectivity/functional network region sets (seen in blue), the language network was the only region set to reach significance, although this did not survive multiple‐comparison correction (*r* = 0.27, *p* = 0.028, *p*FDR = 0.34).

In a previous study using this data, hippocampal morphometry, quantified using the Mahalanobis distance of the hippocampus of interest (HOI), differentiated VNS response groups.^8^ We therefore investigated if HOI and VagAN abnormalities were correlated and whether combining these measures improved prediction of VNS response. VagAN and hippocampal abnormalities were significantly correlated in non‐responders (R=0.41, p=0.0034) but not in responders (R=0.12, p=0.45) or controls (R<0.01, p=0.95, Figure S5). This suggests that the correlation observed in non‐responders reflects epilepsy‐related pathology rather than normal structural variation. A logistic regression combining VagAN and hippocampal abnormalities showed no substantial improvement in response prediction (r=0.32, p=0.009 for the combined measure compared to r=0.30, p=0.008 for the VagAN alone).

## 4 DISCUSSION

Abnormality of the first principal component (PC1) of the VagAN was significantly higher in non‐responders than in responders (r=0.30, p=0.007) and controls (r=0.37, p<0.001). The VagAN showed superior discriminatory value compared with all other region sets tested, including non‐VagAN regions (r=−0.03, p=0.78). In non‐responders, VagAN PC1 abnormality was positively correlated with hippocampal abnormality (R=0.41, p=0.0038), suggesting that non‐response may reflect widespread damage to regions stimulated by VNS. No other region set significantly differentiated outcome groups suggesting that structural abnormalities distinguishing VNS response are relatively specific to the VagAN and the hippocampus, rather than reflecting generalized brain abnormality. It should be noted that the PC1 abnormality score used here represents deviation from normative structural covariance, rather than a marker of any specific pathological feature such as atrophy or hypertrophy. Our findings indicate that the structural organization within the VagAN is more abnormal in non‐responders relative to controls, without implying a uniform directional change across individuals.

The VagAN and hippocampi, comprise key subcortical and cortical regions, are major hubs in brain networks implicated in seizure propagation. VNS is thought to modulate seizure susceptibility through both active electrical stimulation and long‐term neuroplastic functional reorganization.^5^, ^7^, ^30^, ^31^, ^32^ Our findings suggest that structural abnormality in VagAN and hippocampi *before implant* may compromise VNS efficacy by disrupting the neural circuitry that VNS attempts to modulate. While VNS promotes functional “compensation,” it has limited ability to restore structurally degraded tissue,^33^ potentially explaining its limited efficacy in individuals with structural damage in these key areas. We also find an increased likelihood of non‐responders having a lesion visible on MRI which supports this hypothesis; our results hold when only analyzing these individuals (Figure S4) demonstrating that this lesion burden may be indexed towards the VagAN in non‐responders.

The positive correlation between hippocampal and VagAN abnormalities for non‐responders indicates that these abnormalities are not independent but are instead abnormal across VNS‐affected regions (R=0.41, p=0.0038). Comparatively, responders showed no such correlation between abnormalities, demonstrating that responders have more localized abnormalities than non‐responders (R=0.12, p=0.45). Responders' correlation (R=0.12) lies between that of non‐responders (R=0.41) and controls (R<0.01). Similarly, responders' VagAN PC1 abnormality were less abnormal than non‐responders (r=0.30) more closely resembling controls (r=0.05). Taken together, these findings demonstrate that responders' severity of epilepsy in terms of structural damage seen in T1w MRI is relatively low with abnormality values closer to controls than non‐responders. This follows the response hypothesis of maximum abnormality (RHOMA)^5^ which proposed that once a specific “cut‐off point” of abnormality is reached, VNS will be unable to counteract, leading to non‐response.

Interpretation of PC1 VagAN non‐responder abnormality is limited by the amount of variation encapsulated (36.3% in controls). We can state that non‐responders' VagAN structural covariance is abnormal when compared to responders and controls at a high level, but not whether the entire VagAN follows this. PC2 and onward may contain useful information pertaining to VNS response differences, although additional components also explain an ever‐decreasing amount of variation. Therefore, as VNS non‐response has a wide range of causes, these components are too specific to elucidate the large‐scale relationships we are interested in. Differences found in a component with a low variance explanation are challenging to interpret, and testing these in addition increases the chances of incidental findings.

The physiological basis of non‐response likely varies across individuals, encompassing structural, functional, and other factors, necessitating multimodal assessment. In this study, we examined primarily large gray matter structures morphometry. Although relevant for VNS some parts of the VagAN, such as the nucleus tractus solitarius and locus coeruleus were not measured independently due to challenges with their delineation caused by their small size. Additionally, white matter structure, which is quantifiable through diffusion‐weighted imaging (DWI) and fluid‐attenuated inversion recovery (FLAIR) sequences, may also relate to response. Functional imaging modalities, including functional MRI (fMRI), electroencephalography (EEG), and magnetoencephalography (MEG), can capture complementary information about network abnormalities associated with non‐response. Beyond traditional neurophysiological examination, genetic, metabolic, and demographic factors (including factors as wide ranging as handedness^34^) may also influence VNS response. While VagAN abnormality significantly differed between groups, its predictive value for individual people with DRE remains limited due to substantial overlap in the distributions of responders, non‐responders, and controls. This limited discriminatory power suggests that the mechanisms underlying non‐response are heterogeneous, demonstrating the necessity of multimodal assessment.

Response measures are imperfect with an individual's response evolving over time. Some individuals show gradual improvement that may plateau only 5 years post‐implantation,^35^, ^36^ although there are early indications of response.^1^ We determined response from clinical data two‐years post‐implant, which correlates well with long‐term outcomes but does not perfectly predict them. While we took great care in response classification, our measures are subject to the same limitations as patient‐reported seizure diaries, including recall bias and incomplete documentation. Additionally, seizure frequency reduction, while a valuable measure of VNS efficacy, does not fully capture treatment benefit. Some individuals may achieve substantial seizure reduction yet experience limited improvement in quality of life, or conversely, may report improved quality of life with modest seizure reduction (which is not captured in our response classification), although reported improvements in quality of life measures are high amongst individuals who receive VNS.^37^ Differences in response trajectories limit the predictive accuracy of any pre‐implant biomarker, with non‐responder and responder labels only providing a limited description of an individual's complex experience with epilepsy and VNS.

Future studies should seek to clarify whether the effect seen here is site‐specific or generalizes across the larger population of individuals receiving VNS, as recently suggested.^38^ Additionally, work should be done on combining findings across multiple modalities to improve response prediction. Modalities routinely acquired in clinical practice such as EEG, FLAIR, and T1w MRI should be prioritized, as this would maximize generalizability to clinical care. More specifically, combining gray matter volumetric abnormalities with white matter microstructural abnormalities would enhance understanding of VNS non‐response mechanisms. Given that VNS exerts its effects through afferent stimulation throughout the brain, the integrity of both gray matter regions and interconnecting white matter tracts is likely critical for treatment efficacy. Investigating whether concurrent abnormalities in structurally connected regions, especially within the VagAN, influence response represents an important direction for future research.

## AUTHOR CONTRIBUTIONS

Harry J. Clifford: formal analysis and investigation Harry J. Clifford, Peter N. Taylor, and Sonja Fenske: conceptualization and methodology. Sabahat Iqbal and Cameron A. Elliot: data acquisition from hospital records. All authors: interpretation of data for the work, drafting the work or revising it critically for important intellectual content, final approval of the version to be published, and agreement to be accountable for all aspects of the work in ensuring that questions related to the accuracy or integrity of any part of the work are appropriately investigated and resolved.

## FUNDING INFORMATION

TSC is supported by a National Institute for Health and Care Research (NIHR) Newcastle Biomedical Research Centre Fellowship; PNT and YW are supported by UKRI Future Leaders Fellowships (MR/T04294X/1, MR/V026569/1). We are grateful to the Epilepsy Society for supporting the Epilepsy Society MRI scanner. This work was supported by the National Institute for Health Research University College London Hospitals Biomedical Research Centre.

## CONFLICT OF INTEREST STATEMENT

None of the authors has any conflict of interest to disclose.

## ETHICS STATEMENT

We confirm that we have read the Journal’s position on issues involved in ethical publication and affirm that this report is consistent with those guidelines.

## Supporting information


**Figure S1:** Inclusion criteria. Reasons for exclusion from analysis. We collected demographic details of 290 individuals with VNS implantations from the Chalfont Centre for Epilepsy. 198 individuals were excluded due to either; a lack of appropriate T1w MRI scan prior to implantation, large‐scale atrophy or prior resection which make quantitative analysis tools questionable, and lack of ascertainable outcome 2 years after VNS implantation from clinical notes. This left 92 individuals with Epilepsy for analysis (42 Responders and 50 Non‐Responders).
**Figure S2:** PC1 variance & VagAN overlap. Correlation between region count and variance explained by the first principal component (PC1). Each dot represents a region set. We find a negative correlation between the number of regions in a region set and the amount of variance explained by PC1. This correlation is expected and seen when both including VagAN and non‐VagAN (*R* = −0.57, *p* = 0.052) and excluding VagAN and non‐VagAN (*R* = −0.51, *p* = 0.061).
**Figure S3:** Figure 2, excluding individuals with hippocampal sclerosis. VagAN shows greater discriminatory value than all other region sets when excluding individuals with hippocampal sclerosis. VagAN PC1 significantly differentiated non‐responders from responders (*r* = 0.27, *p* = 0.046). The combined non‐VagAN region set showed no discriminatory value (*r* = 0.073, *p* = 0.59), and structural region sets based on lobular anatomy (seen in green) did not significantly differentiate response groups (all *p* > 0.60). Across connectivity/functional network region sets (seen in blue), no network reached significance with the dorsal attention network (DAN) (*r* = 0.25, *p* = 0.061) and language network (*r* = 0.24, *p* = 0.075), only approaching significance. Both the language network and DAN partially overlap with the VagAN (see Table S4).
**Figure S4:** Figure 2, excluding individuals with hippocampal sclerosis and individuals with no clinically identified lesion. VagAN shows greater discriminatory value than all other region sets when excluding individuals with hippocampal sclerosis and individuals with no clinically identified lesion. VagAN PC1 significantly differentiated non‐responders from responders (*r* = 0.41, *p* = 0.042). The combined non‐VagAN region set showed greater abnormality in non‐responders, but this was insignificant (*r* = −0.28, *p* = 0.19). Structural region sets based on lobular anatomy (seen in green) and connectivity/functional network region sets (seen in blue) did not significantly differentiate response groups (all *p* > 0.10).
**Figure S5:** VagAN and hippocampal abnormalities correlate in non‐response with a mild improvement to response prediction. Combined VagAN and hippocampal abnormalities improve response prediction. (A) VagAN PC1 abnormality correlated with hippocampus of interest (HOI) Mahalanobis distance in non‐responders (*R* = 0.41, *p* = 0.0038), but not in responders (*R* = 0.12, *p* = 0.45). (B) A receiver‐operator curve (ROC) for the combined logistic regression model combining VagAN and hippocampal (Area under the curve, AUC = 0.66). (C) Density plots comparing each measure's discriminatory value (combined *r* = 0.32, VagAN PC1 *r* = 0.30, and HOI Mahalanobis *r* = 0.27). Larger differences were observed when comparing controls to both non‐responders (combined *r* = 0.51, VagAN PC1 *r* = 0.37) and responders (combined *r* = 0.16, VagAN PC1 *r* = 0.05).
**Figure S6:** Overlap between region sets. Overlap between region sets. Here, we show the overlap between different region sets, where the overlap percentage is calculated as the number of regions in the intersection of the two sets in question divided by the number of regions in the region set on the *x*‐axis. Mathematically: $\frac{\left|X\cap Y\right|}{|X|}$. For example, the cingulo‐insula represents 11% of the VagAN, while the VagAN represents 50% of the cingulo‐insula.
**Figure S7:** No significant correlation between PC1 variance explained, and biserial *r*. Correlation between absolute biserial *r* and variance explained by the first principal component (PC1). Each dot represents a region set. We find no correlation between the absolute biserial *r* seen in a region set and the amount of variance explained by PC1. This shows that greater explanation of a region set in PC1 does not lead to better differentiation of outcomes to VNS.
**Figure S8:** No significant correlation between number of regions in a region set and biserial *r*. Correlation between absolute biserial *r* and number of regions in a region set. Each dot represents a region set. We find no correlation between the absolute biserial r seen in a region set and the number of regions within it. This shows that larger region counts alone do not help identify response to VNS.
**Figure S9:** PC1 loadings. Loadings used in VagAN PC1 acquired from controls. Almost all VagAN regions show loadings that affect PC1, with the frontal poles being a notable exception, showing little value.
**Data S1:** Summary of lesion type.
**Table S1:** Structural region sets.
**Table S2:** Detailed loadings.
**Table S3:** Mass univariate testing.
**Table S4:** PCA outputs.

## Data Availability

Research data are not shared.
