# Supplementary material for: Structural morphometry distinguishes vagus nerve stimulation outcomes in individuals with epilepsy
Source: Epilepsia Open. 2026 Aug 31;11(5):1755–63. doi: 10.1002/epi4.70339 (PMC13527692; doi:10.1002/epi4.70339)
Supplement: Supplementary file 1 [file EPI4-11-1755-s001.docx]

Structural morphometry distinguishes vagus nerve stimulation outcomes in individuals with epilepsy

Supplementary Materials

Supplementary Figure 1: Inclusion Criteria

**Supplementary Figure 1: Reasons for exclusion from analysis.** We collected demographic details of 290 individuals with VNS implantations from the Chalfont Centre for Epilepsy. 198 individuals were excluded due to either; a lack of appropriate T1w MRI scan prior to implantation, large scale atrophy or prior resection which make quantitative analysis tools questionable, and lack of ascertainable outcome 2 years after VNS implantation from clinical notes. This left 92 individuals with Epilepsy for analysis (42 Responders and 50 Non-Responders)

Supplementary Figure 2: PC1 variance & VagAN overlap

**Supplementary Figure 2: Correlation between region count and variance explained by the first principal component (PC1).** Each dot represents a region set. We find a negative correlation between the number of regions in a region set and the amount of variance explained by PC1. This correlation is expected and seen when both including VagAN and non-VagAN (R=-0.57, p=0.052) and excluding VagAN and non-VagAN (R=-0.51, p=0.061)

Supplementary Figure 3: Figure 2, excluding individuals with hippocampal sclerosis

**Supplementary Figure 3: VagAN shows greater discriminatory value than all other region sets when excluding individuals with hippocampal sclerosis.** VagAN PC1 significantly differentiated non-responders from responders (r=0.27, p=0.046). The combined non-VagAN region set showed no discriminatory value (r=0.073, p=0.59), and structural region sets based on lobular anatomy (seen in green) did not significantly differentiate response groups (all p>0.60). Across connectivity/functional network region sets (seen in blue), no network reached significance with the dorsal attention network (DAN) (r=0.25, p=0.061) and language network (r=0.24, p=0.075), only approaching significance. Both the language network and DAN partially overlap with the VagAN (see supplementary table 4).

# Supplementary Figure 4: Figure 2, excluding individuals with hippocampal sclerosis and individuals with no clinically identified lesion

**Supplementary Figure 4: VagAN shows greater discriminatory value than all other region sets when excluding individuals with hippocampal sclerosis and individuals with no clinically identified lesion.** VagAN PC1 significantly differentiated non-responders from responders (r=0.41, p=0.042). The combined non-VagAN region set showed greater abnormality in non-responders but this was insignificant (r=-0.28, p=0.19). Structural region sets based on lobular anatomy (seen in green) and connectivity/functional network region sets (seen in blue) did not significantly differentiate response groups (all p>0.10).

# Supplementary Figure 5: VagAN and hippocampal abnormalities correlate in non-response with a mild improvement to response prediction

**Supplementary Figure 5: Combined VagAN and hippocampal abnormalities improve response prediction. (A)** VagAN PC1 abnormality correlated with hippocampus of interest (HOI) Mahalanobis distance in non-responders (R=0.41, p=0.0038), but not in responders (R=0.12, p=0.45). **(B)** A receiver operator curve (ROC) for the combined logistic regression model combining VagAN and hippocampal (Area under the curve, AUC=0.66). **(C)** Density plots comparing each measure's discriminatory value (combined r=0.32, VagAN PC1 r=0.30, and HOI Mahalanobis r=0.27). Larger differences were observed when comparing controls to both non-responders (combined r=0.51, VagAN PC1 r=0.37) and responders (combined r=0.16, VagAN PC1 r=0.05).

# Supplementary Figure 6: Overlap between region sets

**Supplementary Figure 6: Overlap between region sets.** Here, we show the overlap between different region sets, where the overlap percentage is calculated as the number of regions in the intersection of the two sets in question divided by the number of regions in the region set on the x-axis. Mathematically: $\frac{\left| X\cap Y \right|}{|X|}$. For example, the cingulo-insula represents 11% of the VagAN, whilst the VagAN represents 50% of the cingulo-insula

# Supplementary Figure 7: No significant correlation between PC1 variance explained, and biserial r

**Supplementary Figure 7: Correlation between absolute biserial r and variance explained by the first principal component (PC1).** Each dot represents a region set. We find no correlation between the absolute biserial r seen in a region set and the amount of variance explained by PC1. This shows that greater explanation of a region set in PC1 does not lead to better differentiation of outcomes to VNS.

# Supplementary Figure 8: No significant correlation between number of regions in a region set and biserial r

**Supplementary Figure 8: Correlation between absolute biserial r and number of regions in a region set.** Each dot represents a region set. We find no correlation between the absolute biserial r seen in a region set and the number of regions within it. This shows that larger region counts alone do not help identify response to VNS.

# Supplementary Figure 9: PC1 Loadings

**Supplementary Figure 9: Loadings used in VagAN PC1** **acquired from controls.** Almost all VagAN regions show loadings that affect PC1, with the frontal poles being a notable exception, showing little value.

# Supplementary Data 1: Summary of lesion type

Individuals had a wide range of aetiologies and pathologies, including hippocampal sclerosis (n=16), focal cortical dysplasia (n=7), glioma (n=4), cyst (n=3), ischemia (n=2), heterotopia (n=2), traumatic brain injury (n=2), encephalitis (n=2), and other pathologies (n=9). 43 individuals had no lesion clinically visible on MRI, and 2 had no specific lesion listed.

# Supplementary Table 1: Structural region sets

| Region set | Contained regions (VagAN, Non-VagAN) |
| --- | --- |
| Cingulate cortex and insula | L & R; Caudalanteriorcingulate, Insula, Rostralanteriorcingulate |
|  | L & R; Entorhinal, Parahippocampal, Posteriorcingulate |
| Frontal lobe | L & R; Caudalmiddlefrontal, Medialorbitofrontal, Parsopercularis, Parstriangularis, Precentral, Rostralmiddlefrontal, Superiorfrontal, Frontalpole |
|  | N/A |
| Occipital lobe | N/A |
|  | L & R; Cuneus, Lateraloccipital, Lingual, Pericalcarine |
| Parietal lobe | L & R; Paracentral |
|  | L & R; Inferiorparietal, Postcentral, Precuneus, Superiorparietal, Supramarginal |
| Temporal lobe | N/A |
|  | L & R; Bankssts, Fusiform, Inferiortemporal, Middletemporal, Superiortemporal, Temporalpole, Transversetemporal |
| Dorsal attention network (DAN) | L & R; Caudalmiddlefrontal |
|  | L & R; Inferiorparietal, Superiorparietal |
| Default mode network (DMN) | L & R; Medialorbitofrontal, Rostralanteriorcingulate |
|  | L & R; Inferiorparietal, Posteriorcingulate, Precuneus |
| Language | L & R; Parsopercularis, Parstriangularis |
|  | L & R; Inferiorparietal, Superiortemporal, Supramarginal |
| Salience | L & R; Caudalanteriorcingulate, Rostralanteriorcingulate, Insula |
|  | N/A |
| Sensorimotor | L & R; Paracentral, Precentral |
|  | L & R; Postcentral |
| Somatosensory | L & R; Paracentral, Insula |
|  | L & R; Postcentral, Supramarginal |
| Visual | N/A |
|  | L & R; Cuneus, Lateraloccipital, Lingual, Pericalcarine |
| VagAN | Brainstem; Pons, Midbrain, Medulla, SCP  L & R; Anterior (hypothalamus), Anterior_Lateral (thalamus), Caudalanteriorcingulate, Caudalmiddlefrontal, Frontalpole, Insula, Intralaminar (thalamus), Lateral_nucleus, Lateralorbitofrontal, Medial (thalamus), Medialorbitofrontal, Paracentral, Parsopercularis, Parsorbitalis, Parstriangularis, Posterior (hypothalamus), Posterior_Central (amygdala), Posterior_Thalamus, Precentral, Rostralanteriorcingulate, Rostralmiddlefrontal, Summed_Nuclei (amygdala), Superiorfrontal, Tubular (hypothalamus), Ventral (thalamus) |
|  | N/A |
| Non-VagAN | N/A |
|  | L & R; bankssts, cuneus, entorhinal, fusiform, inferiorparietal, inferiortemporal, isthmuscingulate, lateraloccipital, lingual, middletemporal, parahippocampal, pericalcarine, postcentral, posteriorcingulate, precuneus, superiorparietal, superiortemporal, supramarginal, temporalpole, transversetemporal |

# Supplementary Table 2: Detailed loadings:

The loadings for VagAN PC1 in descending order are provided below (rounded to 3 decimal places). Only the left_frontalpole demonstrated a negative loading.

| Region | Loading |
| --- | --- |
| Left_Posterior_Central | 0.197 |
| Right_Medial | 0.196 |
| Right_Posterior_Central | 0.192 |
| Left_Medial | 0.188 |
| Right_Summed_Nuclei | 0.187 |
| Left_Summed_Nuclei | 0.185 |
| Left_rostralmiddlefrontal | 0.176 |
| Right_Ventral | 0.174 |
| Midbrain | 0.174 |
| Right_precentral | 0.172 |
| Right_lateralorbitofrontal | 0.169 |
| Left_Tubular | 0.167 |
| Left_Ventral | 0.164 |
| Left_Anterior_Lateral | 0.163 |
| Right_caudalmiddlefrontal | 0.162 |
| Right_Tubular | 0.158 |
| Right_Intralaminar | 0.156 |
| Left_precentral | 0.154 |
| Left_caudalmiddlefrontal | 0.152 |
| Left_Intralaminar | 0.151 |
| Right_Anterior_Lateral | 0.151 |
| Pons | 0.151 |
| Left_lateralorbitofrontal | 0.150 |
| Left_rostralanteriorcingulate | 0.149 |
| Left_Lateral_nucleus | 0.139 |
| Right_superiorfrontal | 0.138 |
| Right_rostralmiddlefrontal | 0.137 |
| Right_Posterior_Thalamus | 0.137 |
| Left_superiorfrontal | 0.134 |
| Left_Posterior_Thalamus | 0.131 |
| Medulla | 0.125 |
| Left_paracentral | 0.122 |
| Left_Anterior | 0.122 |
| Right_Lateral_nucleus | 0.121 |
| Left_insula | 0.117 |
| Right_Anterior | 0.113 |
| Left_parsopercularis | 0.110 |
| Right_Posterior | 0.109 |
| Right_medialorbitofrontal | 0.107 |
| Left_parsorbitalis | 0.103 |
| Right_rostralanteriorcingulate | 0.094 |
| Left_parstriangularis | 0.091 |
| SCP | 0.090 |
| Left_medialorbitofrontal | 0.089 |
| Right_parsorbitalis | 0.087 |
| Left_Posterior | 0.085 |
| Right_parsopercularis | 0.075 |
| Right_caudalanteriorcingulate | 0.064 |
| Left_caudalanteriorcingulate | 0.060 |
| Right_parstriangularis | 0.057 |
| Right_insula | 0.055 |
| Right_paracentral | 0.054 |
| Right_frontalpole | 0.029 |
| Left_frontalpole | -0.013 |

# Supplementary Table 3: Mass univariate testing

The following is the results of mass univariate testing between non-responders and responders using the Wilcoxon rank sum test and rank-biserial correlation in ascending p value order; Raw p = p value without correction, FDR p = p value correction using FDR/FWER, Bon p = p value corrected using Bonferroni correction, Rank biserial = rank-biserial correlation where a negative value indicates less abnormality in responders, AUC = area under the curve

| Region | Raw p | FDR p | Bon p | Rank biserial | AUC |
| --- | --- | --- | --- | --- | --- |
| Left_lateraloccipital | 0.00417 | 0.425 | 0.425 | 0.349 | 0.674 |
| Right_SRLM | 0.0214 | 0.902 | 1.0 | -0.28 | 0.64 |
| Right_parstriangularis | 0.0478 | 0.902 | 1.0 | 0.241 | 0.62 |
| Left_bankssts | 0.0533 | 0.902 | 1.0 | 0.235 | 0.618 |
| Left_Summed_Nuclei | 0.0584 | 0.902 | 1.0 | -0.23 | 0.615 |
| Left_Anterior_Lateral | 0.106 | 0.902 | 1.0 | -0.197 | 0.599 |
| Left_Lateral_nucleus | 0.118 | 0.902 | 1.0 | -0.19 | 0.595 |
| Right_lateralorbitofrontal | 0.12 | 0.902 | 1.0 | 0.19 | 0.595 |
| Right_Lateral_nucleus | 0.127 | 0.902 | 1.0 | -0.186 | 0.593 |
| Left_postcentral | 0.129 | 0.902 | 1.0 | -0.185 | 0.592 |
| Left_supramarginal | 0.135 | 0.902 | 1.0 | -0.182 | 0.591 |
| Right_pericalcarine | 0.137 | 0.902 | 1.0 | 0.181 | 0.59 |
| Left_middletemporal | 0.14 | 0.902 | 1.0 | 0.18 | 0.59 |
| Left_posteriorcingulate | 0.142 | 0.902 | 1.0 | 0.179 | 0.59 |
| Right_lingual | 0.142 | 0.902 | 1.0 | -0.179 | 0.59 |
| Left_Posterior_Central | 0.148 | 0.902 | 1.0 | -0.176 | 0.588 |
| SCP | 0.15 | 0.902 | 1.0 | -0.175 | 0.588 |
| Left_temporalpole | 0.179 | 0.953 | 1.0 | 0.164 | 0.582 |
| Right_parahippocampal | 0.181 | 0.953 | 1.0 | -0.163 | 0.581 |
| Right_caudalanteriorcingulate | 0.197 | 0.953 | 1.0 | 0.157 | 0.579 |
| Right_inferiorparietal | 0.197 | 0.953 | 1.0 | 0.157 | 0.579 |
| Right_CA_DG | 0.232 | 0.953 | 1.0 | -0.146 | 0.573 |
| Left_pericalcarine | 0.241 | 0.953 | 1.0 | 0.143 | 0.571 |
| Right_transversetemporal | 0.244 | 0.953 | 1.0 | -0.142 | 0.571 |
| Right_postcentral | 0.254 | 0.953 | 1.0 | -0.139 | 0.57 |
| Right_precentral | 0.257 | 0.953 | 1.0 | -0.138 | 0.569 |
| Right_superiortemporal | 0.281 | 0.953 | 1.0 | -0.131 | 0.566 |
| Left_cuneus | 0.295 | 0.953 | 1.0 | 0.128 | 0.564 |
| Right_Tubular | 0.333 | 0.953 | 1.0 | -0.118 | 0.559 |
| Right_Summed_Nuclei | 0.337 | 0.953 | 1.0 | -0.117 | 0.559 |
| Right_cuneus | 0.345 | 0.953 | 1.0 | 0.115 | 0.558 |
| Left_rostralmiddlefrontal | 0.361 | 0.953 | 1.0 | 0.111 | 0.556 |
| Left_parahippocampal | 0.395 | 0.953 | 1.0 | -0.104 | 0.552 |
| Left_Medial | 0.404 | 0.953 | 1.0 | 0.102 | 0.551 |
| Left_SRLM | 0.408 | 0.953 | 1.0 | -0.101 | 0.55 |
| Left_Tubular | 0.413 | 0.953 | 1.0 | -0.1 | 0.55 |
| Right_entorhinal | 0.413 | 0.953 | 1.0 | -0.1 | 0.55 |
| Right_Subiculum | 0.426 | 0.953 | 1.0 | -0.0971 | 0.549 |
| Right_precuneus | 0.426 | 0.953 | 1.0 | -0.0971 | 0.549 |
| Left_insula | 0.435 | 0.953 | 1.0 | 0.0952 | 0.548 |
| Right_Posterior | 0.435 | 0.953 | 1.0 | -0.0952 | 0.548 |
| Right_inferiortemporal | 0.435 | 0.953 | 1.0 | -0.0952 | 0.548 |
| Left_entorhinal | 0.44 | 0.953 | 1.0 | 0.0943 | 0.547 |
| Right_medialorbitofrontal | 0.449 | 0.953 | 1.0 | 0.0924 | 0.546 |
| Right_Posterior_Central | 0.459 | 0.953 | 1.0 | -0.0905 | 0.545 |
| Right_fusiform | 0.464 | 0.953 | 1.0 | -0.0895 | 0.545 |
| Left_precuneus | 0.473 | 0.953 | 1.0 | -0.0876 | 0.544 |
| Left_superiorfrontal | 0.488 | 0.953 | 1.0 | -0.0848 | 0.542 |
| Right_middletemporal | 0.493 | 0.953 | 1.0 | 0.0838 | 0.542 |
| Left_parstriangularis | 0.503 | 0.953 | 1.0 | 0.0819 | 0.541 |
| Left_CA_DG | 0.513 | 0.953 | 1.0 | -0.08 | 0.46 |
| Right_Tail | 0.523 | 0.953 | 1.0 | -0.0781 | 0.539 |
| Right_frontalpole | 0.538 | 0.953 | 1.0 | -0.0752 | 0.538 |
| Left_frontalpole | 0.549 | 0.953 | 1.0 | -0.0733 | 0.537 |
| Right_Intralaminar | 0.549 | 0.953 | 1.0 | -0.0733 | 0.537 |
| Left_superiorparietal | 0.549 | 0.953 | 1.0 | -0.0733 | 0.537 |
| Left_rostralanteriorcingulate | 0.57 | 0.953 | 1.0 | 0.0695 | 0.535 |
| Right_insula | 0.57 | 0.953 | 1.0 | 0.0695 | 0.465 |
| Left_Anterior | 0.575 | 0.953 | 1.0 | 0.0686 | 0.466 |
| Right_temporalpole | 0.581 | 0.953 | 1.0 | 0.0676 | 0.534 |
| Left_parsorbitalis | 0.597 | 0.953 | 1.0 | -0.0648 | 0.532 |
| Right_supramarginal | 0.602 | 0.953 | 1.0 | -0.0638 | 0.532 |
| Left_fusiform | 0.608 | 0.953 | 1.0 | 0.0629 | 0.531 |
| Midbrain | 0.635 | 0.953 | 1.0 | 0.0581 | 0.529 |
| Right_isthmuscingulate | 0.641 | 0.953 | 1.0 | 0.0571 | 0.529 |
| Left_lingual | 0.647 | 0.953 | 1.0 | 0.0562 | 0.528 |
| Right_lateraloccipital | 0.647 | 0.953 | 1.0 | 0.0562 | 0.528 |
| Left_isthmuscingulate | 0.652 | 0.953 | 1.0 | 0.0552 | 0.528 |
| Left_Posterior_Thalamus | 0.675 | 0.953 | 1.0 | 0.0514 | 0.526 |
| Left_parsopercularis | 0.686 | 0.953 | 1.0 | -0.0495 | 0.525 |
| Right_Medial | 0.698 | 0.953 | 1.0 | 0.0476 | 0.524 |
| Right_bankssts | 0.721 | 0.953 | 1.0 | 0.0438 | 0.522 |
| Right_rostralmiddlefrontal | 0.727 | 0.953 | 1.0 | 0.0429 | 0.521 |
| Left_inferiortemporal | 0.739 | 0.953 | 1.0 | 0.041 | 0.52 |
| Right_superiorfrontal | 0.745 | 0.953 | 1.0 | 0.04 | 0.52 |
| Medulla | 0.757 | 0.953 | 1.0 | 0.0381 | 0.519 |
| Right_paracentral | 0.775 | 0.953 | 1.0 | -0.0352 | 0.518 |
| Right_Posterior_Thalamus | 0.781 | 0.953 | 1.0 | -0.0343 | 0.483 |
| Right_Anterior | 0.781 | 0.953 | 1.0 | 0.0343 | 0.517 |
| Right_superiorparietal | 0.781 | 0.953 | 1.0 | -0.0343 | 0.517 |
| Left_inferiorparietal | 0.793 | 0.953 | 1.0 | 0.0324 | 0.516 |
| Left_Tail | 0.805 | 0.953 | 1.0 | -0.0305 | 0.515 |
| Left_medialorbitofrontal | 0.811 | 0.953 | 1.0 | 0.0295 | 0.515 |
| Left_lateralorbitofrontal | 0.829 | 0.953 | 1.0 | -0.0267 | 0.513 |
| Right_caudalmiddlefrontal | 0.829 | 0.953 | 1.0 | 0.0267 | 0.513 |
| Left_paracentral | 0.854 | 0.953 | 1.0 | 0.0229 | 0.511 |
| Left_caudalanteriorcingulate | 0.879 | 0.953 | 1.0 | 0.019 | 0.51 |
| Right_Anterior_Lateral | 0.879 | 0.953 | 1.0 | -0.019 | 0.49 |
| Right_parsopercularis | 0.885 | 0.953 | 1.0 | -0.0181 | 0.509 |
| Left_Intralaminar | 0.885 | 0.953 | 1.0 | -0.0181 | 0.509 |
| Pons | 0.885 | 0.953 | 1.0 | 0.0181 | 0.509 |
| Left_Posterior | 0.891 | 0.953 | 1.0 | 0.0171 | 0.491 |
| Left_transversetemporal | 0.897 | 0.953 | 1.0 | -0.0162 | 0.508 |
| Left_precentral | 0.91 | 0.953 | 1.0 | -0.0143 | 0.507 |
| Right_Ventral | 0.91 | 0.953 | 1.0 | 0.0143 | 0.493 |
| Right_posteriorcingulate | 0.91 | 0.953 | 1.0 | -0.0143 | 0.493 |
| Left_Ventral | 0.928 | 0.953 | 1.0 | -0.0114 | 0.494 |
| Left_caudalmiddlefrontal | 0.934 | 0.953 | 1.0 | 0.0105 | 0.495 |
| Left_Subiculum | 0.934 | 0.953 | 1.0 | -0.0105 | 0.505 |
| Left_superiortemporal | 0.934 | 0.953 | 1.0 | -0.0105 | 0.495 |
| Right_rostralanteriorcingulate | 0.984 | 0.991 | 1.0 | -0.00286 | 0.501 |
| Right_parsorbitalis | 0.991 | 0.991 | 1.0 | -0.0019 | 0.501 |

# Supplementary Table 4: PCA Outputs

| Region set | N regions  (N in VagAN) | PC1 Variance Explained | N components explain 80% of variance |
| --- | --- | --- | --- |
| VagAN | 50 (50) | 36.3% | 6 |
| Non-VagAN | 40 (0) | 32.8% | 6 |
| Cingulo-Insula | 12 (6) | 34.7% | 4 |
| Frontal | 20 (20) | 40.0% | 5 |
| Occipital | 8 (0) | 72.1% | 2 |
| Parietal | 12 (2) | 52.0% | 4 |
| Temporal | 14 (0) | 32.8% | 5 |
| DAN | 6 (2) | 53.6% | 2 |
| DMN | 10 (4) | 46.8% | 4 |
| Language | 10 (4) | 36.2% | 4 |
| Salience | 6 (6) | 49.3% | 3 |
| Sensorimotor | 6 (4) | 69.8% | 2 |
| Somatosensory | 8 (4) | 47.2% | 3 |
| Visual | 8 (0) | 72.1% | 2 |
